# RNA markers enable phenotypic test of antibiotic susceptibility in *Neisseria gonorrhoeae* after 10 minutes of ciprofloxacin exposure

**DOI:** 10.1038/s41598-018-29707-w

**Published:** 2018-08-02

**Authors:** Tahmineh Khazaei, Jacob T. Barlow, Nathan G. Schoepp, Rustem F. Ismagilov

**Affiliations:** 1 Division of Biology and Biological Engineering, California Institute of Technology, 1200 E. California Blvd, Pasadena, CA United States of America; 2 Division of Chemistry and Chemical Engineering, California Institute of Technology, 1200 E. California Blvd, Pasadena, CA United States of America

## Abstract

Antimicrobial-resistant *Neisseria gonorrhoeae* is an urgent public-health threat, with continued worldwide incidents of infection and rising resistance to antimicrobials. Traditional culture-based methods for antibiotic susceptibility testing are unacceptably slow (1–2 days), resulting in the use of broad-spectrum antibiotics and the further development and spread of resistance. Critically needed is a rapid antibiotic susceptibility test (AST) that can guide treatment at the point-of-care. Rapid phenotypic approaches using quantification of DNA have been demonstrated for fast-growing organisms (e.g. *E*. *coli*) but are challenging for slower-growing pathogens such as *N*. *gonorrhoeae*. Here, we investigate the potential of RNA signatures to provide phenotypic responses to antibiotics in *N*. *gonorrhoeae* that are faster and greater in magnitude compared with DNA. Using RNA sequencing, we identified antibiotic-responsive transcripts. Significant shifts (>4-fold change) in transcript levels occurred within 5 min of antibiotic exposure. We designed assays for responsive transcripts with the highest abundances and fold changes, and validated gene expression using digital PCR. Using the top two markers (*porB* and *rpmB*) we correctly determined the antibiotic susceptibility and resistance of 49 clinical isolates after 10 min exposure to ciprofloxacin. RNA signatures are therefore promising as an approach on which to build rapid AST devices for *N*. *gonorrhoeae* at the point-of-care, which is critical for disease management, surveillance, and antibiotic stewardship efforts.

## Introduction

*Neisseria gonorrhoeae* is the second most common sexually transmitted bacterial infection in the United States, with about 460,000 cases reported in 2016, an 18.5% rise since 2015^1^. Worldwide, it is estimated that about 78 million new *N*. *gonorrhoeae* infections occur annually^2^. *N*. *gonorrhoeae* infections can lead to heart and nervous system infections, infertility, ectopic pregnancies, newborn blindness, and increased risk for other sexually transmitted infections, including HIV^3^. The CDC has identified *N*. *gonorrhoeae* as one of the three most urgent drug-resistant bacterial threats^3^. *N*. *gonorrhoeae* has developed resistance to all of the most commonly used antibiotics (including penicillins, sulfonamides, tetracyclines, and fluoroquinolones) leaving only one last effective class of antibiotics, cephalosporins. However, there have even been worldwide reported cases of decreased susceptibility to the cephalosporin ceftriaxone^4–8^, and therefore an imminent threat of widespread untreatable *N*. *gonorrhoeae*. An important factor leading to the widespread development of antibiotic resistance is the liberal use and misuse of antibiotics. Critically needed is a rapid antibiotic susceptibility test (AST) that can guide treatment at the point-of-care—both to provide correct treatment and to facilitate antibiotic stewardship.

The gold standard for determining *N*. *gonorrhoeae* susceptibility to antibiotics is the culture-based agar dilution test, which is unacceptably slow (1–2 days). More rapid genotypic approaches, involving detection of gene mutations, are available for a subset of antibiotics in *N*. *gonorrhoeae*^9,10^, but such approaches are inherently limiting, as they require knowledge of the mechanisms of resistance. Moreover, *N*. *gonorrhoeae* is naturally competent for transformation, and can take up gonococcal DNA from the environment and recombine it with its own genome, resulting in frequent gene mutations^11,12^. Given the high rate at which new resistance emerges, relying solely on genotypic methods is not an acceptable long-term solution. Phenotypic methods involving growth measurements have enabled faster ASTs that are independent of resistance mechanisms^13–16^. However, such growth-based methods are challenging for *N*. *gonorrhoeae*, which is slow-growing and fastidious^17^. Another phenotypic approach for antibiotic susceptibility testing is quantification of nucleic acids^18,19^. We have previously demonstrated a rapid (30 min) phenotypic AST using quantification of DNA replication by digital PCR (dPCR) to assess the antibiotic susceptibility of *Escherichia coli* in clinical urine samples^20^. However, AST methods that quantify changes in DNA replication require a longer antibiotic-exposure step for slow-growing pathogens such as *N*. *gonorrhoeae*, which has a doubling time of about 60 min^21^, compared with the 20 min doubling time of *E*. *coli*^22^.

A complementary approach to DNA quantification is measuring the pathogen’s RNA response to antibiotic exposure. Transcriptional responses are among the earliest cellular changes upon exposure to antibiotics^23^, far before phenotypic changes in growth can be observed. Quantifying changes in RNA signatures is therefore a particularly appealing approach for slow-growing organisms. RNA has previously been used to differentiate antibiotic susceptibility and resistance in organisms where the transcriptional response is well characterized^24,25^. More recently, RNA sequencing (RNA-seq) has been used to measure the transcriptome response of *Klebsiella pneumoniae* and *Acinetobacter baumanii* to antibiotic exposure^25^. Although the *N*. *gonorrhoeae* transcriptome has been previously sequenced^26,27^, to our knowledge, no one has characterized the transcriptome response of *N*. *gonorrhoeae* to antibiotic exposure. Unlike most bacteria, *N*. *gonorrhoeae* lacks the classic transcriptional SOS response to DNA damage whereby DNA repair is induced and the cell cycle is arrested^28,29^. The SOS response promotes survival to certain antibiotic classes, such as the fluoroquinolones, which act by directly inhibiting DNA synthesis^30^. The recA or recA-like proteins are essential for the induction of the SOS response^28^. However, neither *recA* transcripts nor recA protein levels increase in *N*. *gonorrhoeae* upon exposure to DNA damaging agents^31,32^.

In this work, we explore the transcriptome response of *N*. *gonorrhoeae* upon exposure to ciprofloxacin. Ciprofloxacin is a fluoroquinolone and functions by inhibiting the enzymes topoisomerase II (DNA gyrase) and topoisomerase IV, thereby inhibiting cell division^33^. Ciprofloxacin was chosen in this study to gain insight into transcriptional changes that occur upon DNA damage in an organism lacking the classic SOS response. Here, we address the following questions: (1) How does the transcriptome of *N*. *gonorrhoeae* respond to ciprofloxacin exposure? (2) What is the shortest antibiotic exposure time at which we can still observe significant changes (>4-fold) in RNA expression? (3) Which transcripts provide the largest and most abundant fold-changes per cell, which is an important consideration for clinical samples that have low numbers of pathogens? (4) Will candidate markers respond consistently across a large pool of isolates with wide genetic variability?

## Results

**Figure 1:**
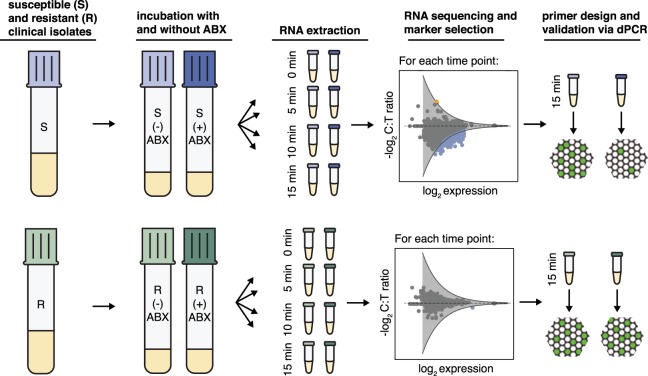
The workflow for selection and validation of RNA markers for phenotypic measurements of antibiotic susceptibility and resistance. Susceptible and resistant isolates of *Neisseria gonorrhoeae* are exposed to antibiotics (ABX) for 5, 10, and 15 min. Samples are collected for RNA sequencing at time zero and every 5 min thereafter. Genes demonstrating fold changes in expression (control:treated ratio (C:T ratio)) greater than the threshold of significance (gray line) are identified as differentially expressed (blue: downregulated and orange: upregulated). Candidate markers are selected from the pool of differentially expressed genes and validated by digital PCR.

We used RNA-seq to study the transcriptome response of susceptible and resistant isolates of *N*. *gonorrhoeae* after 5, 10, and 15 min of ciprofloxacin exposure (Fig. 1). Each clinical isolate culture was initially split into two tubes, where one tube was exposed to the antibiotic (+) and the other served as the control with no antibiotic exposure (−). Samples were collected for RNA-seq prior to antibiotic exposure and every 5 min for 15 min. We calculated the fold change in gene expression between the control and treated samples – defined as the control:treated ratio (C:T ratio); genes that demonstrated significant fold-change differences between the susceptible and resistant isolates were identified as differentially expressed. To account for biological variability, three pairs of susceptible and resistant isolates were used in this study. Candidate markers were selected from the pool of differentially expressed genes and were validated using droplet dPCR (see Methods).

### Temporal shifts in global gene expression upon antibiotic exposure

**Figure 2:**
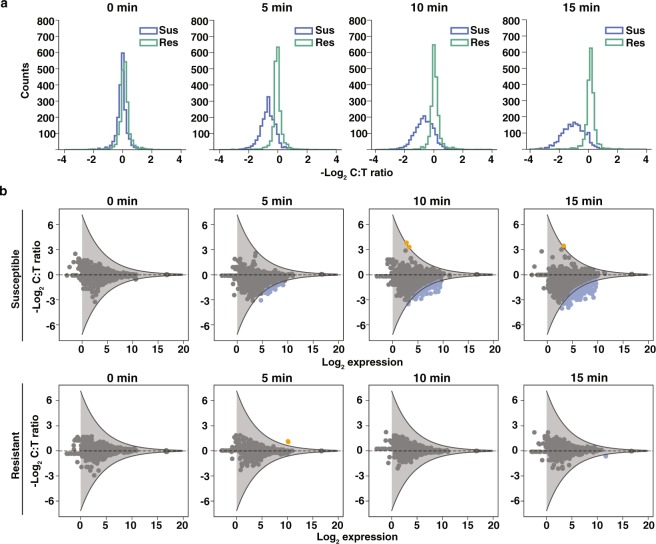
Temporal shifts in global gene expression upon ciprofloxacin exposure in *Neisseria gonorrhoeae*. (**a**) The distribution of −log_2_(C:T ratios) for a susceptible isolate (Sus) and resistant isolate (Res) at 0, 5, 10, and 15 min. (**b**) The fold change in gene expression between control and treated samples (C:T ratio) versus expression in the control sample at 0, 5, 10, and 15 min for one susceptible isolate and one resistant isolate. Genes with C:T ratios above or below the significance threshold are identified as differentially expressed (blue: downregulated; orange: upregulated). Thresholds for statistical significance of fold change (gray lines) are determined by fitting a negative exponential curve (with 90% confidence interval) to the outer edge of the −log_2_ C:T ratios measured at time zero (see Methods).

We observed global shifts in RNA expression in susceptible isolates in as early as 5 min after antibiotic exposure (Fig. 2a). The distribution of fold changes in gene expression levels (C:T ratios) indicated global shifts toward negative log_2_ fold-change values (downregulation). The magnitude of fold change at which most genes were distributed was approximately 2-fold. The tail of the distribution illustrates that a few genes responded to antibiotic exposure with changes as large as 6-fold within 5 min. Increasing the antibiotic exposure time further shifted the distribution to larger negative log_2_ fold-change values. The transcriptional response in resistant isolates was tightly distributed around a fold-change value of 1 at every time point, indicating that the transcriptome of the resistant isolates did not respond significantly to antibiotic exposure (Fig. 2a).

To identify genes that were differentially expressed between control and treated samples, we defined a threshold of significance (Fig. 2b). The threshold of significance took into account technical variability and was calculated from the C:T ratios at t = 0 min of all biological replicates that were sequenced (three susceptible and three resistant isolates). For each of the six gene expression datasets (one for each isolate), we plotted the −log_2_(C:T ratio) against the −log_2_(expression) for all genes and fit a negative exponential curve to the outer edge of each plot. We then averaged the curves from all six datasets and added a 90% confidence interval to the average curve by assuming a Gaussian fit for the error distribution, which we define as our threshold of significance. Genes with a −log_2_(C:T ratio) value above or below the upper and lower thresholds were identified as differentially expressed. Downregulated genes (fold changes below the significance threshold) appeared as early as 5 min after antibiotic exposure (blue dots, Fig. 2b). Two upregulated genes (fold changes above the significance threshold) appeared after 10 min of exposure (orange dots, Fig. 2b).

### Selection of candidate markers that are consistent in response and abundant

**Figure 3:**
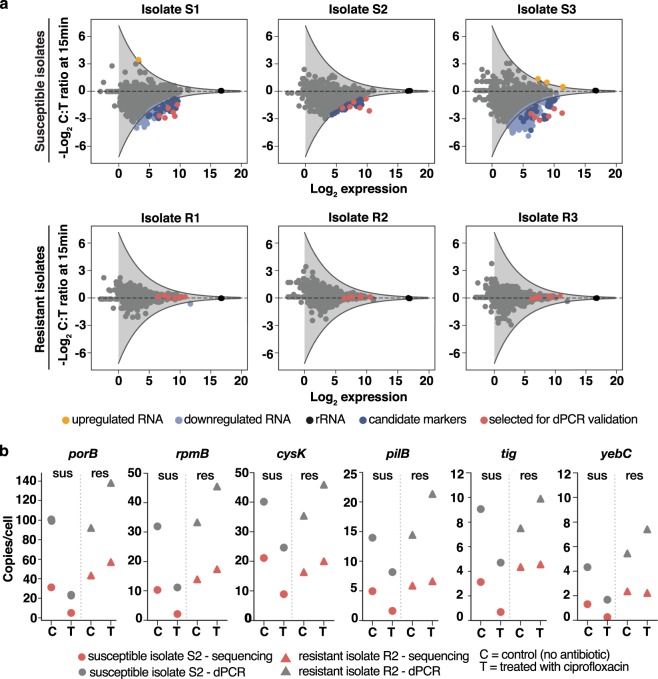
Selection of candidate RNA markers for phenotypic antibiotic susceptibility testing in *Neisseria gonorrhoeae* and measurements of candidate marker abundances per cell (**a**) Genes that are differentially expressed (light blue) across three pairs of resistant and susceptible clinical isolates are identified as candidate markers (dark blue). Six candidate markers that span different biological functions were selected for validation (red). (**b**) Copies/cell values for the candidate markers are determined from RNA sequencing (red) and dPCR (gray) (see Methods). Data is shown for one pair of susceptible (S2) and resistant (R2) isolates at 15 min of ciprofloxacin exposure.

RNA expression in response to antibiotics can be heterogeneous among different isolates of the same species^34^; thus, it is important to select candidate markers from differentially expressed genes that respond consistently across isolates of *N*. *gonorrhoeae*. To identify these candidate markers, we exposed three different pairs of susceptible isolates (minimum inhibitory concentrations (MICs) <=0.015 mg/mL) and resistant isolates (MICs 2.0 mg/mL, 4.0 mg/mL, and 16.0 mg/mL) to ciprofloxacin for 15 min and extracted RNA for sequencing (see workflow in Fig. 1). We found 181, 41, and 410 differentially expressed genes in susceptible isolates 1, 2, and 3, respectively (Fig. 3a). Among the differentially expressed genes, 38 genes responded consistently across the three pairs of susceptible and resistant isolates (i.e. responses overlapped in all three susceptible isolates, whereas all three resistant isolates were non-responsive) (Supplementary Table S1). These genes spanned a variety of biochemical functions in the cell. We selected six candidate transcript markers for further analysis based on the following criteria: (1) high fold change; (2) high expression levels (>75 transcripts per million, TPM); and (3) representative of different biochemical pathways. The selected candidate markers were: *porB* (membrane protein), *rpmB* (ribosomal protein), *tig* (molecular chaperone), *yebC* (transcriptional regulator), *pilB* (pilus assembly ATPase), and *cysK* (cysteine synthase). The candidate marker with the highest abundance and largest fold change upon antibiotic exposure was *porB*, which is a membrane channel forming protein and the site of antibiotic influx into the cell^35^.

A high level of gene expression was one of our criteria for selection of candidate markers from the sequencing data. High expression of candidate markers is not only important for sensitivity and limits of detection, but is particularly important for clinical samples with low numbers of pathogen cells. One of the advantages of RNA compared with DNA as a nucleic acid marker is its natural abundance in the cell. Because the gene expression values obtained from sequencing are relative values, our next step was to quantify the absolute copies per cell for the candidate markers. In our quantification approach, we plated clinical isolate samples after 15 min of ciprofloxacin exposure to obtain cell numbers in colony forming units (CFU/mL). We designed primers for the candidate markers (see Methods and Supplementary Table S2) and measured their absolute concentration using dPCR. The concentrations were converted to per-cell values using the cell counts from plating (Fig. 3b). Additionally, we used the RNA sequencing data to obtain transcriptome-wide estimates of transcript copies per cell. In the sequencing approach, we added external RNA control consortium (ERCC) spike-ins to the lysis buffer step of the extraction protocol in order to capture any loss of RNA throughout the extraction steps. There are potential artifacts associated with the ERCC approach^36^. By linear regression we captured the relationship between ERCC copies added to the samples and ERCC quantified by sequencing. Using the linear regression, we converted gene expression values from RNA sequencing (in TPM) to approximate copy numbers per cell (see Methods). The transcript copies per cell estimated for the candidate markers using the sequencing approach were within the same order of magnitude as the absolute copies per cell measured by dPCR (Fig. 3b).

### Validation of candidate markers by dPCR

**Figure 4:**
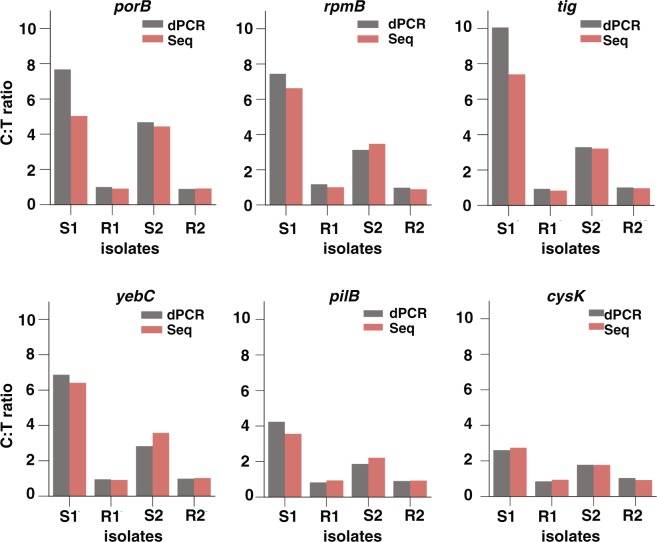
Validation of the RNA sequencing approach using digital PCR (dPCR) with six candidate markers. Control:treated ratios (C:T ratios) determined by RNA sequencing (red) were validated against C:T ratios measured by dPCR (gray). The dPCR C:T ratios were normalized using ribosomal RNA (rRNA) by dividing the C:T ratio of the candidate marker by the C:T ratio of 16S rRNA. This normalization step is not required for sequencing data because the values are normalized by sequencing depth (see Methods). Markers were validated using two susceptible (S1 and S2) and two resistant (R1 and R2) isolates at 15 min of ciprofloxacin exposure.

We next asked how the relative changes observed through RNA-seq compare with direct gene expression measurements by dPCR. We designed dPCR assays for candidate markers, which involved measuring the absolute expression of the candidate marker in both control and treated samples, and calculating the C:T ratio. In this assay, the 16S rRNA was also measured and used to normalize the C:T ratio of the candidate markers. In the three susceptible isolates that were sequenced we found that rRNA consistently showed the smallest fold change (<1.06) in response to ciprofloxacin compared with all other genes in *N*. *gonorrhoeae*. Therefore, to account for experimental variations in the antibiotic exposure and RNA extraction steps between control and treated samples, we used the 16S rRNA as an intracellular control for normalizing the C:T ratios (see Methods). We found that the C:T ratios measured by the dPCR assay agreed with the C:T ratios obtained through sequencing (Fig. 4), confirming that both approaches accurately capture the transcriptional response to antibiotic exposure.

### Validation of RNA markers across CDC isolates

**Figure 5:**
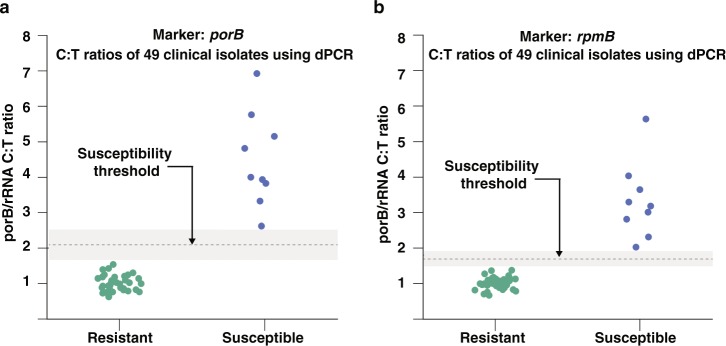
Antibiotic susceptibility testing of 49 clinical isolates using (**a**) *porB*, and (**b**) *rpmB* as RNA AST markers. Antibiotic susceptibility of 49 clinical isolates (9 susceptible and 40 resistant) from the *Neisseria gonorrhoeae* panel of the CDC Antimicrobial Resistance Isolate Bank was determined using the “normalized” C:T ratios (C:T ratio of marker/C:T ratio of 16S rRNA). Clinical isolates were exposed to ciprofloxacin for 10 min and the concentration of RNA markers was measured by digital PCR.

Finally, we asked whether candidate markers respond consistently across a large pool of isolates with genetic variability. We chose the two candidate markers with the highest abundances and fold changes (*porB* and *rpmB*) to determine the susceptibility of 49 clinical isolates, with a wide range of MIC values (Supplementary Table S3), from the *N*. *gonorrhoeae* panel of the Centers for Disease Control (CDC) Antimicrobial Resistance Isolate Bank. The MIC values were representative of the population-wide distribution values reported by the European Committee on Antimicrobial Susceptibility Testing^37^. We exposed each clinical isolate to ciprofloxacin for 10 min and measured the fold change in expression of the two candidate markers between the control and treated sample using dPCR (Fig. 5). Both markers correctly classified all 49 CDC isolates, based on Clinical and Laboratory Standards Institute (CLSI) breakpoint values, as 9 susceptible and 40 resistant strains.

## Discussion

In this work, we demonstrate that antibiotic-responsive transcripts can be used as suitable markers for a rapid phenotypic AST in *N*. *gonorrhoeae*.

When characterizing the global transcriptional response of *N*. *gonorrhoeae* to antibiotic exposure, we observed a significant change in response in as early as 5 min. The nature of the response was a global downregulation in transcript levels. Among the candidate markers, all exhibited downregulation in response to ciprofloxacin. We specifically looked at *gyrA* and *parC*, which are known genotypic markers of resistance to ciprofloxacin, and differential expression was not observed. We also looked at the *recA* transcript because *recA* is one of the prominent genes in the SOS response. As expected, because *N*. *gonorrhoeae* does not have a true SOS system^28,29^, we did not find *recA* levels to increase. Whereas *recA* is a specific cellular response to overcome DNA damage, the global downregulation that we observed suggests a general shift away from growth and cell proliferation. Among the 38 candidate markers, 15 were ribosomal proteins (including one of the top markers, *rpmB*), which play a prominent role in assembly and function of the ribosomes and are essential for cell growth. Mutations in ribosomal proteins have been reported to confer resistance to different classes of antibiotics^38^. Another top marker identified in this study was *porB*, which is a membrane channel forming protein (porin) responsible for uptake of small nutrients and the site of antibiotic influx into the cell. The expression of porins is highly regulated in response to environmental stimuli^39^. Reducing permeability to decrease intracellular antibiotic concentration is a known mechanism for bacteria to confer antibiotic resistance^38^. The downregulation of *porB* observed in this study can be attributed to a halt in growth processes caused by ciprofloxacin damage and possibly an attempt to reduce influx of antibiotic.

A key aim of this study was to identify RNA markers that would yield a measurable response after only a short antibiotic exposure (<15 min) to ensure this approach can fit within the required timescale for a rapid AST. It is possible that longer exposure times could provide additional insight into the biological response of *N*. *gonorrhoeae* to ciprofloxacin, but this was not the focus of our study. Furthermore, the short exposure times potentially introduce a bias in selection of transcripts present at low abundances. For transcripts present at high abundance to display the same fold change as low abundance transcripts, a substantially higher number of mRNA molecules must be transcribed, which would require longer timescales. As an example, a 4-fold change from 1 to 4 transcripts requires 3 additional mRNA to be produced, whereas a 4-fold change from 20 to 80 requires 60 mRNA to be transcribed. This bias also holds true in downregulation, where mRNA continues to be transcribed in the control samples, whereas transcript levels drop in treated samples due to degradation of RNA, and/or a reduction in the rate of transcription.

We identified candidate markers with consistent differential expression across three sets of susceptible and resistant pairs. Among the candidate markers, one of our criteria for selection was transcript abundance, which is of particular importance in clinical samples with low cell numbers. Furthermore, marker abundance affects measurement sensitivity and limits of detection, as has been previously demonstrated in AST methods based on quantification of DNA replication^20^. To measure the abundance of the candidate markers, we used both dPCR measurements and ERCC spike-ins for RNA sequencing to obtain approximate RNA copies/cell. Both methods yielded results within the same order of magnitude. To our knowledge this is the first quantitative measurement of RNA abundance per cell in *N*. *gonorrhoeae*.

We separately validated the performance of the two most abundant candidate markers, *porB* and *rpmB*, with 49 clinical isolates. Both markers were consistent in their ability to correctly determine susceptibility or resistance of all 49 clinical isolates. *porB* demonstrated C:T ratios between 2.5 to 7 and *rpmB* demonstrated C:T ratios between 2 and 6 after 10 min of antibiotic exposure in the nine susceptible clinical isolates. The large fold changes highlight the significance of using RNA response as an AST marker compared with quantification of DNA replication. Our previous work using dPCR quantification of DNA replication demonstrated C:T ratios between 1.2 and 2.4 for 15 min of antibiotic exposure in *E*. *coli*^20^, which has a doubling time approximately 3 times shorter than *N*. *gonorrhoeae*.

We performed an alignment search of *porB* against other prokaryotes and found it to be specific to the *Neisseria* genus. AST markers should be specific to the pathogen of interest because additional bacterial species are likely to be present in clinical samples. Additional experiments with mixtures of bacteria would be required to further confirm the specificity of the markers identified in this study. We additionally measured the 16S rRNA to normalize C:T ratios, which inherently enables pathogen identification as well. A combination of identification and susceptibility testing in a single integrated platform is important for correct and rapid diagnosis.

This paper demonstrates that RNA markers can be used to determine antibiotic susceptibility of *N*. *gonorrhoeae* after short antibiotic exposure times, a requirement for a rapid phenotypic AST. *N*. *gonorrhoeae* is a fastidious slow-growing organism, presenting challenges to growth-based AST methods. Additional work will be needed to yield a clinic-ready, rapid RNA-based AST for *N*. *gonorrhoeae*. Additional background matrices of clinical samples, both urine and swab samples, that could possibly affect speed and sensitivity of an AST, must be further evaluated. Digital isothermal chemistries, such as digital loop-mediated isothermal amplification (dLAMP) should be considered to speed up quantification times relevant to point-of-care settings^20^. Follow-up studies should also examine the transcriptional response of *N*. *gonorrhoeae* to other classes of antibiotics and identify responsive RNA markers for class-specific antibiotics. Overall, as a first step, the work described here demonstrates the potential of a phenotypic RNA-based approach for a rapid AST in *N*. *gonorrhoeae* at the point-of-care, which is critically needed for disease management, surveillance, and antibiotic stewardship.

## Methods

### Antibiotic exposure for RNA sequencing

Antibiotic susceptible and resistant clinical isolates were obtained from the University of Washington Neisseria Reference Laboratory and the University of California, Los Angeles, Clinical Microbiology Laboratory. Isolates were plated from glycerol stocks onto Chocolate Agar plates and grown in static incubation overnight (37 °C, 5% CO_2_). Cells were re-suspended in Hardy Fastidious Broth (HFB) and incubated for 45 min (37 °C, 5% CO_2_) with shaking (800 rpm) to an OD_600_ between 1 and 5. Cultures were diluted (5X) into HFB. Each isolate culture was split into “treated” and “control” tubes. Ciprofloxacin was added to the “treated” tubes (final concentration of 0.5 µg/mL) and water was added to the “control” tubes; cultures were incubated (static; 37 °C, 5% CO_2_) for 15 min. During incubation, samples were collected for RNA sequencing at 5, 10, and 15 min (300 µL aliquot of sample was mixed into 600 µL of Qiagen RNA Protect Reagent (Qiagen, Hilden, Germany) for immediate RNA stabilization). In addition, a sample was collected for RNA sequencing immediately before ciprofloxacin was added. To quantify CFU, the sample at t = 15 min was serially diluted (10X), plated on a Chocolate Agar plate, and incubated overnight (37 °C, 5% CO_2_).

### Antibiotic exposure for clinical isolates

Antibiotic susceptible and resistant clinical isolates were obtained from the *N*. *gonorrhoeae* panel of the CDC Antimicrobial Resistance Isolate Bank. Isolates were plated from glycerol stocks onto Chocolate Agar plates and grown in static incubation overnight (37 °C, 5% CO_2_). Cells were re-suspended in pre-warmed HFB + 5 mM sodium bicarbonate and incubated for 30 min (37 °C, 5% CO_2_) with shaking (800 rpm) to an OD_600_ between 1 and 5. Cultures were diluted (100X) into HFB + 5 mM sodium bicarbonate. Each isolate culture was split into treated (0.5 µg/mL final concentration of ciprofloxacin) and control (water instead of antibiotic) samples. Samples were incubated at 37 °C for 10 min on a static hot plate. A 90 µL aliquot of each sample was placed into 180 µL of Qiagen RNA Protect Reagent for immediate RNA stabilization. A 5 µL aliquot of each sample was plated onto a Chocolate Agar plate and incubated overnight (37 °C, 5% CO_2_) as a control for the exposure experiments. If the expected growth phenotypes (i.e. resistant = growth; susceptible = no growth) were not observed for any single sample in the plating control, the exposure experiment was repeated for the set of samples. From the 50 total isolates available from the *N*. *gonorrhoeae* panel of the CDC Antimicrobial Resistance Isolate Bank, 49 were used in this study. One isolate was excluded from this study because we suspected that it had been contaminated; we did not detect *porB* primer amplification using qPCR.

### RNA sequencing and analysis

RNA was extracted using the Enzymatic Lysis of Bacteria protocol of the Qiagen RNeasy Mini Kit and processed according to the manufacturer’s protocol. DNA digestion was performed during extraction using the Qiagen RNase-Free DNase Set. The quality of extracted RNA was measured using an Agilent 2200 TapeStation (Agilent, Santa Clara, CA, USA). Extracted RNA samples were prepared for sequencing using the NEBNext Ultra RNA Library Prep Kit for Illumina (New England Biolabs, Ipswitch, MA, USA) and the NEBNExt Multiplex Oligos for Illumina. Libraries were sequenced at 50 single base pair reads and a sequencing depth of 10 million reads on an Illumina HiSeq. 2500 System (Illumina, San Diego, CA, USA) at the Millard and Muriel Jacobs Genetics and Genomics Laboratory, California Institute of Technology. Raw reads from the sequenced libraries were subjected to quality control to filter out low-quality reads and trim the adaptor sequences using Trimmomatic (version 0.35). The reads were aligned to the FA 1090 strain of *N*. *gonorrhoeae* (NCBI Reference Sequence: NC_002946.2) using Bowtie2 (version 2.2.5) and quantified using the Subread package (version 1.5.0-p1). A pseudocount of 1 was added to the gene quantification; gene expression was defined in transcripts per million (TPM). In this study, tRNA was excluded from the sequencing data.

### Marker selection

For each gene, we defined the C:T ratio as the gene expression (TPM) in the control sample divided by the gene expression (in TPM) in the treated sample. We plotted the −log_2_(C:T) against the −log_2_(expression in TPM) for all genes. To identify genes that were differentially expressed between control and treated samples, we defined a threshold of significance. The threshold of significance was calculated from the C:T ratios at t = 0 min for the biological replicates that were sequenced (three susceptible and three resistant isolates). For each of the six gene expression datasets (one for each isolate), we fit a negative exponential curve to the outer edge of each plot and then averaged the curves from all six datasets. Finally, we added a 90% confidence interval to the average curve by assuming a Gaussian fit for the error distribution, which is our threshold of significance. Genes with a −log_2_(C:T) value above or below the upper and lower thresholds were identified as differentially expressed. Genes that were differentially expressed consistently (either always above or always below the thresholds) among the three susceptible isolates and were not differentially expressed among the three resistant isolates were defined as candidate markers.

### Copies/cell measurements from sequencing

To measure copies per cell using sequencing data, we added 2uL of (1/1000 dilution) ERCC RNA Spike-In Mix (Thermo Fisher Scientific, Waltham, MA, USA) to the lysis buffer in the RNeasy Mini Kit to each individual sample. We calculated the number of copies of each ERCC transcript in the sample, by accounting for dilution and multiplying by Avogadro’s number (manufacturer’s concentrations were reported in attomoles/µL). We plotted the relationship between log_2_(ERCC copies added) against log_2_(gene expression in TPM) and performed a linear regression in the region of linearity. We used the linear regression to convert TPM values to total RNA copies in each sample. Finally, using the CFU measured for each sample from plating (described in the “Antibiotic exposure for RNA sequencing” section), the total RNA copies were converted to copies per cell.

### Validation with droplet digital PCR (dPCR)

Primers were designed for candidate markers using Primer-BLAST^40^ and primer alignments were verified using SnapGene. Expression of candidate markers was quantified using the Bio-Rad QX200 droplet dPCR system (Bio-Rad Laboratories, Hercules, CA, USA). The concentration of the components in the dPCR mix used in this study were as follows: 1 × EvaGreen Droplet Generation Mix (Bio-Rad), 150 U/mL WarmStart RTx Reverse Transcriptase, 800 U/mL RiboGaurd RNase Inhibitor, 500 nM forward primer, and 500 nM reverse primer. The RNA extraction comprised 5% of the final volume in the dPCR mix. The remaining volume was nuclease-free water. For each isolate, candidate marker expression was quantified in the control and treated samples and the fold-change difference (C:T ratio) was calculated. To account for potential differences between the control and treated samples that could arise from experimental variability and extraction efficiency, we used ribosomal RNA (rRNA) as an internal control because from our sequencing data, we found that rRNA was not affected by antibiotic exposure in the time frame of this study. To normalize by rRNA, we quantified the 16S rRNA in the control and treated samples by dPCR and calculated an rRNA C:T ratio. We then divided the C:T ratio of each marker by the rRNA C:T ratio. All dPCR C:T ratios reported in this paper are the normalized C:T ratios.

### Data availability

Data on candidate markers for *N*. *gonorrhoeae* and sequences for the primers used in this study are provided in the Supplementary file available. The complete sequencing data generated during this study are available in the National Center for Biotechnology Information Sequence Read Archive repository under the study accession number SRP150785.

## Electronic supplementary material

Supplementary Information
