# Supplementary material for: RNA markers enable phenotypic test of antibiotic susceptibility in Neisseria gonorrhoeae after 10 minutes of ciprofloxacin exposure
Source: Sci Rep. 2018 Aug 2;8:11606. doi: 10.1038/s41598-018-29707-w (PMC6072703; doi:10.1038/s41598-018-29707-w)
Supplement: Supplementary file 1 — Supplementary Information [file 41598_2018_29707_MOESM1_ESM.pdf]

Supplementary Information

RNA markers enable phenotypic test of antibiotic susceptibility in *Neisseria gonorrhoeae* after 10 minutes of ciprofloxacin exposure

Tahmineh Khazaei[1], Jacob T. Barlow[1], Nathan G. Schoepp[2], and Rustem F. Ismagilov*[1,2]
[1] Division of Biology and Biological Engineering, California Institute of Technology
[2] Division of Chemistry and Chemical Engineering, California Institute of Technology
1200 E. California Blvd., Pasadena, CA, United States of America
*Correspondence to: rustem.admin@caltech.edu

Supplementary Table S1. List of candidate markers and their expression in transcripts per million (TPM) and copies per cell for susceptible isolate S2 and resistant isolate R2 after 15 min of ciprofloxacin exposure. The genome used for alignment was *N. gonorrhoeae* FA1090 (NCBI Reference Sequence: NC_002946.2).

		Susceptible (S2) Control		Susceptible (S2) Treated		Resistant (R2) Control		Resistant (R2) Treated	
**Locus Tag**	**Gene Description**	**TPM**	**copies/cell**	**TPM**	**copies/cell**	**TPM**	**copies/cell**	**TPM**	**copies/cell**
NGO0340	cysteine synthase A (*cysK*)	894.1	21.1	505.2	8.9	551.8	16.3	600.0	20.0
NGO1837	50S ribosomal protein L4 *(rplD)*	474.9	10.8	262.2	4.4	403.6	11.9	425.4	13.8
NGO1843	elongation factor G (fusA)	433.4	9.8	224.9	3.8	432.9	12.8	503.5	16.6
NGO2024	50S ribosomal protein L13 (*rplM*)	415.0	9.4	213.5	3.6	455.3	13.5	503.5	16.6
NGO1845	30S ribosomal protein S12 (*rpsL*)	563.1	13.0	286.8	4.9	615.4	18.2	697.6	23.5
NGO1677	50S ribosomal protein L27 (rpmA)	410.7	9.3	192.2	3.2	500.6	14.8	497.6	16.4
NGO1844	30S ribosomal protein S7	520.0	11.9	241.3	4.0	520.1	15.4	651.6	21.9
NGO0171	50S ribosomal protein L19 (*rplS*)	379.2	8.5	175.0	2.9	328.5	9.7	353.2	11.3
NGO1834	30S ribosomal protein S19 (*rpsS*)	330.0	7.4	152.1	2.5	260.9	7.7	292.7	9.2
NGO0172	tRNA (guanine-N(1)-)-methyltransferase (*trmD*)	237.3	5.2	108.8	1.7	208.8	6.2	224.6	6.9
NGO1835	50S ribosomal protein L2 (*rplB*)	392.5	8.9	179.1	2.9	297.6	8.8	359.8	11.5
NGO1673	type IV pilus assembly protein (*pilB*)	225.9	4.9	101.5	1.6	199.3	5.9	214.9	6.6
NGO1833	50S ribosomal protein L22 (*rplV*)	343.8	7.7	147.9	2.4	292.1	8.6	304.3	9.6
NGO2173	50S ribosomal protein L32 (*rpmF*)	407.5	9.2	173.6	2.9	394.7	11.7	404.1	13.1
NGO0604	30S ribosomal protein S1 (*rpsA*)	437.9	9.9	185.3	3.1	456.3	13.5	493.9	16.2
NGO0016	preprotein translocase subunit (*secG*)	180.1	3.9	73.7	1.1	169.1	5.0	184.5	5.6
NGO2174	hypothetical protein	372.8	8.4	150.2	2.4	368.3	10.9	361.6	11.6
NGO2164	GMP synthase (guaA)	118.3	2.5	45.0	0.7	98.6	2.9	109.4	3.2
NGO1676	50S ribosomal protein L21 (*rplU*)	554.6	12.8	200.4	3.3	555.2	16.4	587.7	19.6
NGO1679	50S ribosomal protein L33 (*rpmG*)	283.8	6.3	101.4	1.6	298.5	8.8	284.3	8.9
NGO1658	hypothetical protein	98.4	2.1	33.8	0.5	118.3	3.5	116.1	3.4
NGO1440	macrolide transport protein MacA	143.3	3.1	48.6	0.7	132.3	3.9	139.7	4.2
NGO0174	30S ribosomal protein S16 (*rpsP*)	315.2	7.0	101.2	1.6	295.8	8.7	340.5	10.9
NGO0173	ribosome maturation factor RimM (*rimM*)	359.8	8.1	113.5	1.8	316.8	9.4	318.8	10.1
NGO0592	trigger factor (*tig*)	146.5	3.1	45.5	0.7	147.5	4.3	152.1	4.6
NGO1680	50S ribosomal protein L28 (*rpmB*)	452.8	10.3	130.3	2.1	470.2	13.9	525.4	17.3
NGO0620	aspartate alpha-decarboxylase	64.8	1.3	18.6	0.3	54.2	1.6	59.3	1.7
NGO1659	intracellular septation protein A	62.2	1.3	17.8	0.3	63.6	1.9	70.7	2.0
NGO1291	transcriptional regulator (yebC)	64.1	1.3	18.0	0.3	79.9	2.3	77.9	2.2
NGO0648	membrane protein	56.4	1.1	15.3	0.2	47.6	1.4	45.2	1.2
NGO0593	ATP-dependent Clp protease proteolytic subunit (*clpP*)	60.2	1.2	16.0	0.2	73.6	2.2	75.9	2.2
NGO1804	(3R)-hydroxymyristoyl-ACP dehydratase (*fabZ*)	91.0	1.9	24.0	0.3	74.6	2.2	73.5	2.1
NGO0618	membrane protein	81.4	1.7	20.1	0.3	66.8	2.0	70.2	2.0

NGO0619	2-dehydro-3-deoxyphosphooctonate aldolase	61.1	1.2	15.1	0.2	51.1	1.5	62.6	1.8
NGO1812	major outer membrane protein (*porB*)	1293.2	31.2	293.4	5.0	1459.1	43.3	1587.1	57.1
NGO1890	glutamate permease; sodium/glutamate symport carrier protein	35.0	0.7	7.5	0.1	40.3	1.2	48.9	1.3
NGO2098	diaminopimelate decarboxylase	26.0	0.5	4.9	0.1	18.6	0.5	18.6	0.5
NGO2100	frataxin-like protein (*cyaY*)	20.4	0.4	3.6	0.0	14.0	0.4	18.1	0.5

Supplementary Table S2. Primer sequences used for validation of candidate markers by digital PCR.

**Candidate Marker**	**Gene Name**	**Forward Primer Sequence**	**Reverse Primer Sequence**
porB	major outer membrane porin	GCTACGATTCTCCCGAATTTGCC	CCGCCKACCAAACGGTGAAC
rpmB	50S ribosomal protein L28	TTGCCCAACTTGCAATCACG	AGCACGCAAATCAGCCAATAC
tig	trigger factor	AAAGCCTTGGGTATTGCGG	TGACCAAAGCAACCGGAAC
yebC	YebC/PmpR family Transcriptional Regulator	GCTTTGGAAAAAGCAGCCG	GGTTTTGTTGTCGGTCAGGC
pilB	Type IV-A pilus assembly ATPase	GACTTTTGCCGCTGCTTTG	GCGCATTATTCGTGTGCAG
cysK	Cysteine synthase A	GAGGCTTCCCCCGTATTGAG	TTCAAAAGCCGCTTCGTTCG
16S rRNA	16S ribosomal RNA	ACTGCGTTCTGAACTGGGTG	GGCGGTCAATTTCACGCG

Supplementary Table S3. Minimum inhibitory concentration **(**MIC) values for the 49 *Neisseria gonorrhoeae* clinical isolates acquired from the CDC and FDA Antibiotic Resistance Isolate Bank[1].

**MIC**	**Number of strains**	**Susceptible or Resistant**
0.015	8	Susceptible
0.03	1	Susceptible
4	1	Resistant
8	6	Resistant
16	33	Resistant

[1] CDC and FDA Antibiotic Resistance Isolate Bank. Atlanta (GA): CDC. (2018)
